# Biological Properties of JNK3 and Its Function in Neurons, Astrocytes, Pancreatic β-Cells and Cardiovascular Cells

**DOI:** 10.3390/cells9081802

**Published:** 2020-07-29

**Authors:** Rei Nakano, Tomohiro Nakayama, Hiroshi Sugiya

**Affiliations:** 1 Laboratory for Cellular Function Conversion Technology, RIKEN Center for Integrative Medical Sciences (IMS), 1-7-22 Suehiro-cho, Tsurumi-ku, Yokohama 230-0045, Japan; 2 Laboratory of Veterinary Radiology, College of Bioresource Sciences, Nihon University, 1866 Kameino, Fujisawa 252-0880, Japan; nakayama.tomohiro80@nihon-u.ac.jp (T.N.); sugiya.hiroshi@nihon-u.ac.jp (H.S.)

**Keywords:** JNK3, intracellular signaling, physiological function, cellular reprogramming

## Abstract

JNK is a protein kinase, which induces transactivation of c-jun. The three isoforms of JNK, JNK1, JNK2, and JNK3, are encoded by three distinct genes. JNK1 and JNK2 are expressed ubiquitously throughout the body. By contrast, the expression of JNK3 is limited and observed mainly in the brain, heart, and testes. Concerning the biological properties of JNKs, the contribution of upstream regulators and scaffold proteins plays an important role in the activation of JNKs. Since JNK signaling has been described as a form of stress-response signaling, the contribution of JNK3 to pathophysiological events, such as stress response or cell death including apoptosis, has been well studied. However, JNK3 also regulates the physiological functions of neurons and non-neuronal cells, such as development, regeneration, and differentiation/reprogramming. In this review, we shed light on the physiological functions of JNK3. In addition, we summarize recent advances in the knowledge regarding interactions between JNK3 and cellular reprogramming.

## 1. Introduction

Mitogen-activated protein kinase (MAPK) signaling pathways regulate various cellular functions [1,2,3,4,5]. MAPKs, which act as Ser-Thr kinases, include c-Jun NH_2_-terminal kinase (JNK), p38 MAPK, and extracellular signal-regulated kinase (ERK). Several isoforms of all MAPKs are present in mammals. The mechanisms underlying the activation of MAPKs depend on the stimulus and cell type. MAPK activation results in specific cellular responses via phosphorylation of a wide range of substrates such as transcription factors and cytoskeletal proteins [1,2]. MAPK signaling cascades consist of three hierarchically sequential kinase components: a MAPK kinase kinase (MAPKKK); a MAPK kinase (MAPKK); and a MAPK. MAPKKKs activate MAPKKs by phosphorylating Ser or Thr residues, which subsequently induce the activation of MAPKs via phosphorylation of both Thr and Tyr residues in its activation loop [6,7].

JNK has been described as a protein kinase, which induces the transactivation of c-jun by phosphorylating N-terminal Ser-63 and Ser-73 residues [8,9]. JNK activation requires dual phosphorylation of Tyr and Thr residues. MAPKKs, MKK4, and MKK7 are required for JNK phosphorylation. Although the biochemical properties of MKK4 and MKK7 are different, both MKK4 and MKK7 are considered as upstream regulators of JNK signaling. MKK4 and MKK7 reportedly induce the activation of JNK via dual phosphorylation of Tyr and Thr residues [10,11]. MKK4 induces phosphorylation of both JNK and p38 MAPK, whereas MKK7 specifically induces activation of JNK [12]. Several MAPKKKs were reported as upstream regulators of MKK4 and MKK7: apoptosis-regulating kinases (ASKs); mixed lineage protein kinases (MLKs); and dual leucine zipper kinases (DLKs) [13,14,15]. Some scaffold proteins have also been reported to regulate JNK signaling [16].

Three isoforms of JNK, JNK1 (also known as JNK-1 kinase, p46, or SAPKγ), JNK2 (also known as SAP kinase, p54, or SAPKα) and JNK3 (also known as p493F12 kinase or SAPKβ), are encoded by three distinct genes. JNK genes are located on different chromosomes. JNKs are alternatively spliced to form 10 JNK isoforms [17]. The functional differences between splice variants remain unclear, but different tissues express distinct subsets of JNK isoforms, suggesting that JNK activation plays a crucial role in cellular function. Reportedly, JNKs are required for embryonic development, cellular proliferation, apoptosis, and multiple cellular processes in differentiated cells [18]. JNK1 and JNK2 are expressed ubiquitously throughout the entire body. It is well known that isoforms JNK1 and JNK2 play different roles in regulating c-Jun expression and cell proliferation [19,20]. By contrast, the expression pattern of JNK3 is limited and observed mainly in the brain, heart, and testes [18]. Genomic cloning revealed that *JNK3* consists of 14 exons and 13 introns. The transcription-initiation site and the termination codon are located at exon 3 and exon 14, respectively [21]. In mice, these isoforms are *Jnk1* on chromosome 14 B, *Jnk2* on chromosome 11 B1.3, and *Jnk3* on chromosome 5, whereas in humans these correspond to chromosomal locations of 10q11.22, 5q35, and 4q21.3, respectively [22]. The objective of the current review was to summarize the role and intracellular signaling of JNK3 in the development, regeneration and differentiation/reprogramming in neuronal lineage cells and non-neuronal cells (e.g., astrocytes, pancreatic β-cells, cardiovascular cells).

## 2. Biological Properties of JNK3

JNK3 was first reported by Mohit et al. as a cytoplasmic antigen identified via the monoclonal antibody 3F12 and named p493F12 kinase [23]. Martin et al., reported that p493F12 kinase belongs to a subfamily of MAP kinases, which includes JNK1 and JNK2 [24,25]. This subfamily, known as the JNK family, exhibits high amino acid identity and the kinases belonging to this family are more distantly related to other MAP kinases, with a 40–45% identity. Alternative splicing of JNK3, p46 JNK3α1 and p54 JNK3α2 has been reported.

### 2.1. Structure of JNK3

The crystal structure of unphosphorylated JNK3 (p46 JNK3α1), adenylyl imidodiphosphate and magnesium complex has been described at a resolution of 2.3 å [26]. JNK3 has a typical kinase fold, with the ATP-binding site situated within a cleft between the N- and C-terminal domains. The ATP-binding site of JNK3 is well ordered, wherein the glycine-rich nucleotide-binding sequence forms a *β*-strand–turn–*β*-strand structure over the nucleotide. Unphosphorylated JNK3 assumes an open conformation, in which the N- and C-terminal domains are twisted apart relative to their positions in cAMP-dependent protein kinase. Such rotation leads to the misalignment of some of the catalytic residues. The phosphorylation lip of JNK3 partially blocks the substrate-binding site.

Hydrogen/deuterium exchange mass spectrometry revealed that the C-terminal tail is largely intrinsically disordered. The conformation of the kinase domain of p54 JNK3α2 is more dynamic than that of p46 JNK3α1. Differences in conformation dynamics between long and short splice variants of JNK3α may affect the cellular functions of JNK3 [27].

The dynamics and the energetics of JNK3 in unphosphorylated, phosphorylated, and ATP-bound phosphorylated states have been reported [28]. Unphosphorylated JNK3 undergoes ‘open-to-closed’ movement via a two-step mechanism. Phosphorylation and ATP-binding allow JNK3 kinase to attain a fully active conformation.

### 2.2. Downstream Target Proteins of JNK3

JNK protein kinases were initially identified as kinases that bind and phosphorylate Ser 63 and Ser 73 residues in the NH_2_-terminal activation domain of c-Jun [29,30]. Phosphorylation of the Ser 73 residue in c-Jun increases transcriptional activity [8,9,31]. In addition to c-Jun, ATF2 and Elk-1 transcription factors are phosphorylated in Chinese hamster ovary (CHO) cells overexpressing JNK isoforms. However, in vitro binding studies revealed that it was c-Jun and ATF2, and not Elk-1, that bound to full-length JNKs, suggesting that binding and phosphorylation occur separately, which may be attributed to in vitro interaction between JNK and substrates [17]. These observations suggest that the expression of multiple JNK isoforms, including JNK3, may involve a mechanism that generates tissue-specific responses by activating the JNK signal transduction pathway.

The tumor suppressor protein p53 (Ser 34) was also identified as a substrate of JNK3 [32]. Transfection of Human embryonic kidney cells 293 (HEK-293T) cells with a C-terminal FLAG-tagged JNK3 cDNA expression vector (FLAG-JNK3 cDNA) alone led to the phosphorylation of p53 (Ser-34). Furthermore, strong phosphorylation of Ser-34 in p53 was observed in HEK-293T cells co-transfected with FLAG-JNK3 cDNA and HPK1 cDNA or MEKK1 cDNA. Therefore, JNK3 may play an important role in nuclear signal transduction in response to environmental stress factors or tumorigenic agents, via p53 Ser-34 phosphorylation.

The mitogen-activated kinase activating death domain/differentially expressed in neoplastic vs. normal cells protein, which is implicated in the pathogenesis of Alzheimer’s disease, was identified as a specific substrate of JNK3, although the precise residue involved remains unknown [33,34].

Superior cervical ganglion-10 (SCG10, Stathmin-2) is a membrane-associated protein found in growth cones of neurons. SCG10, which regulates microtubule dynamics and phosphorylation, is implicated in microtubule-destabilizing activity and transduction of cell surface signals to microtubules. Protein complex kinase assays and tandem mass spectrometry analyses have revealed that Ser-73 and Ser-62 residues in SCG10 were phosphorylated by p54 JNK3α2 [35].

Co-immunoprecipitation of PLC-γ1 and JNK3 indicated that these two proteins may directly bind to each other. Incubation of JNK3 with recombinant PLC-γ1 and PLC-γ2 in the presence of [γ-32P] ATP revealed that the PLC-γ1 (D-domain) was an in vitro substrate of JNK3. Thus, suggesting that phosphorylation of recombinant PLC-γ1 was induced by JNK3 [36].

### 2.3. Downstream Target Genes of JNK3

Differential gene expression in the hippocampus of JNK3 knockout mice (6-week-old males) was examined via microarray [37]. Twenty-two differentially expressed transcripts (z-score > 2) including 5 RIKEN cDNA and 17 known proteins were obtained, where 10 were upregulated and 7 were downregulated. The mRNA and protein expression of p110β was increased in JNK3 null mice. Increased phosphorylation of Akt (Ser-473) and GSK3β (Ser-9), which are downstream targets of p110β, was also observed. These observations suggested that JNK3 regulates the activation of the PI3K/Akt signaling pathway.

### 2.4. Endogenous Stimulator for JNK3

Triggers of JNK3 activation were reported by studies investigating HA-tagged JNK3 expressed in small cell lung cancer cell line Shadyside hospital pittsburgh-77 cells (SHP77) and rat pheochromocytoma cell line PC12 [38]. Ultra violet (UV) irradiation induced the activation of JNK3. Treatment with 300 mM mannitol, which stimulated osmotic stress, promoted levels of activated JNK3 that were similar to levels achieved with UV. Treatment of HA-tagged p46 JNK3-expressing SHP77 cells with 600 mM mannitol induced activation of JNK3 to levels twice that induced by UV. In contrast, UV and osmotic stress specifically activated HA-tagged JNK expression in PC12 cells. Only p46 JNK3, p54 JNK3, and JNK2α were activated by UV irradiation and 300 mM mannitol; the mechanism underlying this process remains unclear. These observations suggested that the 10 JNK isoforms may have distinct functions, leading, in turn, to specific responses in diverse tissues and cell types.

Angiotensin II peptide activates p54 JNK3α2 via the angiotensin AT1a receptor [39]. In CV-1 origin SV40 (COS-7) cells expressing the AT1a receptor, a slight phosphorylation of p54 JNK3α2 was induced following treatment with angiotensin II peptide. These observations suggested that a G protein coupled receptor may stimulate the activation of p54 JNK3α2 [40]. AT1a receptor couples to Gq/11, which induces activation of phospholipases and an increase in [Ca^2+^]_i_. Since the increase in [Ca^2+^]_i_ plays an important role in the regulation of intracellular signaling and cellular function (e.g., cell growth, differentiation and secretary function), [Ca^2+^]_i_ and Ca^2+^/calmodulin-dependent protein kinase II (CaMKII) are considered to be involved in the JNK3 activation, but the interaction remains to be elucidated.

In mouse fibrosarcoma L929 cells, hyaluronidase induced the activation of JNK, and this activation was inhibited by dominant negative JNK1, JNK2, and JNK3 [41].

### 2.5. Regulatory Proteins Upstream of JNK3

Activation of JNK3 was tightly regulated by MAPK/ERK (MEK) kinase, an upstream regulator belonging to the MKK protein family. HEK 293-T cells were transfected with a plasmid expressing GST-p54 JNK3α2 and variable amounts of plasmids expressing untagged MKK4 (also known as MAP2K4, SEK1). Low levels of MKK4 expression increased specific kinase activity of GST-p54 JNK3α2, relative to that of GST-p54 JNK3α2 isolated from cells transfected with p54 JNK3α2 alone, suggesting that MKK4 acts as a direct activator of p54 JNK3α2. On the other hand, higher levels of MKK4 expression resulted in the inhibition of p54 specific kinase activity, indicating that MKK4 acts as an inhibitor of p54 JNK3α2 kinase activity, when present in equimolar or greater quantities. The N-terminal segment of MKK4, including residues 35-69, is necessary for the inhibition of p54 JNK3. MKK6 (also known as MAP2K6, SEK3, XMek3) efficiently activated p54 JNK3α2 in addition to p38. Further, high concentrations of MKK6 did not inhibit p54 JNK3 [42].

MKK7 also acts as an upstream regulator of p46 JNK3α1 [43]. Activation of p46 JNK3α1 in vitro was induced by MKK4, MKK7, and MKK4/7. MKK4 induced phosphorylation of the Tyr residue in p46 JNK3α1, whereas MKK7 had no effect. However, MKK4-induced phosphorylation of Tyr in p46 JNK3α1 was enhanced in the presence of MKK7. A combination of MKK4 and MKK7 induced bisphosphorylation of p46 JNK3α1, but neither MKK4 nor MKK7 treatment alone induced a significant increase in such bisphosphorylation. The sequence of the phosphorylated peptide was TAGTSFMMT(PO_3_)PY(PO_3_)VVTR. In the presence of MKK7 or MKK4/7, the maximal velocity (*V*max) increased. MMK7-induced phosphorylation of Thr-221 in p46 JNK3α1 was sufficient to activate p46 JNK3α1. MMK4 phosphorylated p46 JNK3α1 on Tyr-223 following MMK7-induced Thr phosphorylation.

Synergistic activation of p46 JNK3α1 by the combination of MKK4 and MKK7α was confirmed in Sf21 insect cells-expressing p46 JNK3α1 (∆1–38) [44]. MKK4 failed to induce the phosphorylation of p46 JNK3α1 at 0.01 μM, but was able to phosphorylate Tyr-185 following the phosphorylation of Thr-183 by MKK7. Amino acid sequencing confirmed that both peptides correspond to residues 175–190, where MKK4 is phosphorylated at Tyr-185 and MKK7α at Thr-183. In addition to MKK7α, MKK7β was reported to activate p46 JNK3α1, at a rate which was approximately 500-fold faster than that of MKK7α [44]. Considered together, these results suggested that both MKK4 and MKK7α/β are required for full phosphorylation of the p46 JNK3α1 activation loop.

The binding site of MKK4 was identified using an in vitro binding assay [45]. Full-length and N-terminal truncation mutants of MKK4-(37–399) bound to GST-JNK3, whereas N-terminal truncation mutants of MKK4-(47–399) failed to bind to GST-JNK3, suggesting that residues 37–46, which contain the putative JNK-docking site and the first anthrax lethal factor cleavage site, are necessary for MKK4/JNK3 binding. All JNK isoforms, including JNK3, were able to bind to GST-MKK4-(1–94), but failed to bind to GST-MEK1-(1–60) or GST-MEK2-(1–64). MKKs specifically bind to their cognate MAPKs via their N-terminal domains.

Interaction between p54 JNK3α2, MKK4 and MKK7 has been reported [41]. Although whether MKK4/7 directly binds to p54 JNK3α2 was unclear. MKK4, but not MKK7, bound to β-arrestin 2, the scaffold protein for JNKs, in COS-7 cells co-expressing FLAG-β-arrestin 2, GST-MKK4 and GST-MKK7. Interaction between MKK4 and β-arrestin 2 was enhanced in the presence of exogenous p54 JNK3α2 or ASK1.

The interaction of p54 JNK3α2 with MKK4 and MKK7 was examined in COS-7 cells expressing GST-MKK4, GST-MKK7 and p54 JNK3α2. MKK4 and MKK7 induced phosphorylation of Tyr and Thr residues, respectively, in p54 JNK3α2 [46].

Activation of MAPKs is also regulated via dephosphorylation by Tyr phosphatases, Ser/Thr phosphatases and dual specificity phosphatases (DSPs, also known as MKPs). JNKs were regulated by the specific DSPs, MKP7, M3/6 (hVH5), and MKP5. Transfection of COS-7 cells with HA-JNK3, GST-β-arrestin 2 and ASK1, with or without MKP-7, demonstrated that MKP-7 inhibited JNK3 activation in the presence of β-arrestin 2, suggesting that MKP-7 attenuates activation of the pool of JNK3 bound to β-arrestin 2 [47].

### 2.6. Scaffold Protein for JNK3 Activation

#### 2.6.1. JNK-Binding Proteins

The yeast two-hybrid system was applied to search for JNK3 binding proteins, in order to elucidate the process by which JNK3 response maintains its specificity, [48,49]. JNK-binding protein JNKBP1, a mouse homolog of KIAA0596, and JNK/SAPK-associated protein 1 (JSAP-1, JIP3) were identified. JNKBP1, which is ubiquitously expressed in many tissues, is highly expressed in the brain. The JNK-binding region of JNKBP1, which is located between residues 1063 and 1331, interacted with JNK1, JNK2, and JNK3, while less binding was observed with other MAP kinases. JNKBP1 showed a higher binding affinity to JNK2 compared to JNK1 or JNK3. Overexpression of full-length JNKBP1 activated JNK but had no effect on other MAP kinases. However, in the presence of MEKK1 and TAK1, JNKBP1 further enhanced MEKK1 or TAK1-induced JNK activation. Since JNKBP1 does not appear to contain a kinase domain, it is unlikely that JNKBP1 directly activates the component(s) of JNK cascades via phosphorylation. Thus, it is surmised that JNKBP1 may play a scaffolding role via binding to multiple components of the JNK signaling pathway, including JNKs, MAPKK (MKKs) and MAPKKKs [48].

JNK/SAPK-associated protein 1 (JSAP-1, JIP3) is also a protein binding to JNK3 [49,50]. Northern blotting revealed that mouse JSAP1 was expressed almost exclusively in the brain. JSAP1 was also detected in the cytoplasm of retinoic acid-induced differentiated P19 cells. The specificity of JSAP1 binding affinity to MAP kinases was examined via co-transfection experiments. COS-7 cells were co-transfected with His-S-tagged full-length JSAP1 and either Flag epitope-tagged JNK1, JNK2, JNK3, ERK2, or p38α. While JNK1, JNK2, and JNK3 were detected in the precipitated fraction, no bands representing ERK2 and p38a were detected. Among JNKs, JNK3 dominantly binds to JSAP1. Binding between in vitro-translated 35S-labeled JNK3 and a series of Trx-His-S-JSAP1 fusion proteins containing various portions of JSAP1 was examined to confirm the region of JSAP1 that interacts with JNK. The results indicated that the JNK-binding region was located between residues 201 and 217 in JSAP1, which was encoded by a region of exon 6. Four isoforms of JSAP1, JSAP1a, JSAP1b, JSAP1c and JSAP1d, were generated by alternative splicing variants involving exons 5 and 6. Although all JSAP1 isoforms possess the JNK binding domain, JSAP1c and JSAP1d have a 31-aa sequence, which is not observed in JSAP1a, or JSAP1b. Since JSAP1c and JSAP1d showed a lower binding affinity for JNK3, it is most likely that JSAP1c and JSAP1d inhibit the scaffolding activity of JSAP1a and JSAP1b in JNK [51].

In HEK293 cells transfected with Myc-JSAP1, JSAP1 formed a complex with MKK4, MKK7 and JNK3. When HEK293 cells were co-transfected with WT-ASK1 these interactions were enhanced, but the catalytically inactive mutant of ASK1 exerted no effect. In vitro phosphorylation analysis revealed that GST-JSAP1 was phosphorylated by a constitutively active form of ASK1 GST-ASK1ΔN, but not by the inactive kinase mutant GST-ASK1ΔN-KR. GST-JSAP1 (744–1305) was directly phosphorylated by ASK1, but not by JNK3, suggesting that ASK1-induced phosphorylation of JSAP1 contributes to JNK activation [52].

#### 2.6.2. β-Arrestin 2

β-arrestin 2, also known as arrestin 3, is reportedly a binding partner of JNK3 [39,53,54,55,56,57]. A yeast two-hybrid system based on GAL4–β-arrestin 2 fusion protein, used to screen rat brain cDNA libraries, identified the C-terminal portion of p54 JNK3α2 as a binding partner of β-arrestin 2 [39]. Endogenous interaction between β-arrestin 2 and p54 JNK3α2 in mouse brains was confirmed via immunoprecipitation using anti-β-arrestin 2 antibodies. It was found that p54 JNK3α2 was more abundant in the β-arrestin 2-precipitated fraction, while p46 JNK3α1 was not. In COS-7 cells expressing FLAG-β-arrestin 2, HA-ASK1, HA-MKK4 and HA-p54 JNK3α2, β-arrestin 2 enhanced ASK1-induced phosphorylation of p54 JNK3α2, but left phosphorylation of MKK4 unaffected, suggesting that β-arrestin2 was required to activate signaling related to phosphorylation of JNK3. The carboxyl terminus of β-arrestin 2 was identified as the region that interacts with p54 JNK3α2. This region encodes Lys-Pro (KP) in β-arrestin1 and Arg-Arg-Ser (RRS) in β-arrestin 2. Although wild type β-arrestin 2 binds to p54 JNK3α2, binding of mutant β-arrestin 2 (RRS residues replaced with KP) to p54 JNK3α2 is reduced [58,59]. Reportedly, Val-343 was the key residue involved in binding, whereas Leu-278, Ser-280, His-350, Asp-351, His-352 and Ile-353 played a supporting function. Binding of β-arrestin 2 to ASK1 or JNK3 did not correlate with β-arrestin 2-induced ASK1-dependent JNK3 phosphorylation [60].

The presence of β-arrestin 2 does not alter the specificity of MKK4 and MKK7 for target amino acid residues [61,62]. ASK1-induced p54 JNK3α2 activation depends on endogenous MKK4 and MKK7, which were unaffected by overexpression of ASK1, JNK3α2 and β-arrestin 2. Direct binding of β-arrestin 2 with both MKK4 and MKK7 was demonstrated via an in vitro pulldown assay using purified protein. Whereas p54 JNK3α2 enhanced the binding of β-arrestin 2 to MKK4, it reduced the binding of β-arrestin 2 and MKK7. Competition experiments associated with the in vitro pulldown assay, indicated that β-arrestin 2 prevented MKK4 and MKK7 from forming hetero-oligomers. It was also observed that increasing MKK7 reduced the amount of MKK4 bound to MBP-β-arrestin 2, suggesting that the binding sites for MKK4 and MKK7 overlapped.

Since β-arrestin 2 was identified as a partner of heterotrimeric guanine nucleotide binding protein-coupled receptor function, the function of β-arrestin 2 was examined in COS-7 cells expressing the AT1a receptor [40]. Angiotensin II peptide induced the activation of p54 JNK3α2, which was enhanced in COS-7 cells co-expressing FLAG-β-arrestin 2. Localization of FLAG-β-arrestin 2 was predominantly cytoplasmic. GFP-p54 JNK3α2 indicated that p54 JNK3α2 was localized in the cytosol as well as in the nucleus. Co-transfection of COS-7 with FLAG-β-arrestin 2, GFP-p54 JNK3α2 and HA-ASK1 revealed that ASK1-activated p54 JNK3α2 was distributed in the cytosol.

In HEK 293 cell expressing FLAG-β-arrestin 2, GFP-p54 JNK3α2, and AT1A receptor, β-arrestin 2 and p54 JNK3α2 were localized in the cytoplasm. A portion of p54 JNK3α2 was translocated to the nuclei of cells treated with angiotensin II peptide, but nuclear p54 JNK3α2 remained in its inactive form. Phosphorylated p54 JNK3α2 was localized in endosomal vesicles with β-arrestin 2. A point mutation of the nuclear export signal (L395A) in β-arrestin 2, induced the redistribution of β-arrestin 2 to the nucleus and caused nuclear localization of JNK3. These data indicated that subcellular distribution of JNK3 was regulated by nucleocytoplasmic shuttling of β-arrestin 2 [63].

The interaction between AT1R, β-arrestin 2 and p54 JNK3α2 was confirmed by an in vitro spin-down, bioluminescence resonance energy transfer assay of intact cells as well as co-immunoprecipitation. These experiments showed that JNK3α2 interacted with the non-receptor-binding side of β-arrestin 2. The 25-amino acids of the N-domain element showed the highest affinity for p54 JNK3α2, suggesting that it acted as the key site for p54 JNK3α2 docking [63].

The β1 strand of β-arrestin 2 constitutes the major site that interacts with JNK3. C-lobe regions near the activation loop of JNK3 form a potential binding interface, which varies depending on ATP binding status. Because the β1 strand of β-arrestin 2 is covered by the C-terminal strand in its basal state, C-terminal truncation (i.e., pre-activation) of β-arrestin 2 facilitates the β-arrestin 2/JNK3 interaction [64].

A conveyor belt model based on a combined experimental and computational approach was proposed for understanding JNK3 activation and β-arrestin 2 scaffolding [65]. An active JNK3 is exchanged for an inactive JNK3 on the β-arrestin 2/MKK complex leading to signal amplification. In addition, the model of scaffold-dependent signal amplification is conceptually applicable to other scaffold-dependent signaling pathways.

### 2.7. Palmitoylation in JNK3

Protein palmitoylation, which is a mode of post translational modification, regulates protein trafficking and functions by reversibly attaching the lipid, palmitate, to cysteine (Cys) residues. Palmitoyl acyl transferases (PATs) catalyze palmitoylation depending on protein sequence and specificity. The Cys residues required for palmitoylation are located at the COOH-termini of p54 JNK3α1, but not in that of p54 JNK3α2. Primary cortical or hippocampal neuronal cultures from embryonic days 18–19 incubated with ^3^H-palmitate for 3 h, exhibited ^3^H-palmitate incorporation into JNK3, but not into JNK1 or JNK2. The palmitoylation inhibitors, 2-bromopalmitate and hydroxylamine, prevented palmitoylation of JNK3. In HEK293 cells expressing several neuronal PATs (zD15, zD20 and zD23), the PATs, zD15 and zD20, significantly induced JNK3 palmitoylation. Point-mutation of one or both Cys residues (JNK3 CS) to Ser prevented PAT zD15-induced palmitoylation. These results suggest that the JNK3 was the dominant palmitoylated isoform in neurons [66].

### 2.8. Other Proteins Interacting with JNK3

JNK3 was identified as a potential protein binding partner of p16INK4a in a mammalian two-hybrid system. Exposure to UVC increased the interaction between p16INK4a and JNK3. In SK-MEL-5 (p16INK4a-null) cells co-transfected with GST-p16INK4a and V5-tagged JNK3, p16INK4a interacted with JNK3, suggesting that a specific high-affinity interaction occurred between p16INK4a and JNK3. JNK3 deletion mutant analysis revealed that the N-terminal region of JNK3, including the glycine-rich site, is necessary for binding p16INK4a. The Global Range Molecular Matching calculation used for docking simulation experiments of p16INK4a and JNK3 suggest that p16INK4a binds to the N-terminal domain (S1) of JNK3, which contains the glycine-rich loop. The hydrogen-bond networks between β-strands in the N-terminal domain of JNK3 and the ankyrin repeats of p16INK4a play a crucial role in binding. Arg-58 and Glu-88 in p16INK4a form charge to charge interactions with Asp-87 and Lys-68 in JNK3, respectively. Hydrogen bonding between the polar residues in the ankyrin repeats of p16INK4a (Arg-24, Arg-58 and Met-53) and in the N-terminal domain of JNK3 (Tyr-82, Asp-87 and Asn-89) contributed to the specificity and stability of the p16INK4a–JNK3 complex [67].

A comprehensive analysis of the binding partners of JNK1α1 and JNK3α1 was performed using the yeast two-hybrid system [68]. JNK1-specific interactors (ATF7, FUS, KCNE4, PIAS1, SHANK1, TKT), typical JNK-binding domain-dependent interactors shared by JNK1α1 and JNK3α1 (AKAP6, BMPR2, EEF1A1, GFAP, GRIP2, GTF2F1, HDAC2, MAP1B, MYO9B, PTPN2, RABGAP1, RUSC2, SUMO1, SYPL1, TOPBP1, ZNF668), or JNK3-specific partners (ATXN1, NNAT, PTGDS) were identified based on interaction with the JNK3 N-terminal extension.

### 2.9. Regulation of JNK3 mRNA Expression Level

Regulation of JNK3 mRNA expression at the promoter level has been reported (Yoshida et al., 2003, Ying et al., 2006). *Fas-associated phosphatase-1* (*FAP-1*) is located 633 bp upstream of *JNK3*. A short G/C-rich region is located between the cap sites of *JNK3* and *FAP-1,* which contains a bidirectional promoter region for *JNK3* and *FAP-1* including multiple cis elements (Sp1, AP-1, AP-2, GATA-1, a GC box, and a CCAAT box) [69]. Tumor suppressor functions of *JNK3* and *FAP-1* were frequently epigenetically disrupted in multiple lymphomas and carcinomas via the methylation of their shared bidirectional promoter [70].

The PI3K/Akt signaling has been shown to play important roles in cellular function [71]. PI3K/Akt signaling induces the phosphorylation of the mammalian target of rapamycin complex1 (mTORC1 Ser-2448), which contributes viability, regulation of cytoskeletal rearrangement, membrane expansion, transcription, and translation. In Parkinson’s disease (PD) rat model induced by continuous subcutaneous injection of low dose rotenone, which exhibited typical phenotypes related to PD; occasional paralysis of limbs, inconvenience of walking, inability to eat, gradual loss of ability to resist arrest, yellowing of hair and bow back. Although the role of JNK3 in PD models has to be elucidated, the expression of JNK3 mRNA and protein in brains were decreased in the presence of the PI3K inhibitor LY294002 and mTORC1 inhibitor rapamycin [72].

## 3. Physiological Role and Cellular Signaling of JNK3

The in vivo function of JNK3 has been studied in mice and rats. In addition, cellular models were also useful for checking cascade and signaling interactions of JNK3 in vitro. Involvement of JNK3 in certain physiological events of neuronal cells and tissues has been reported. Moreover, the involvement of JNK3 in pathophysiological events, such as stress response or cell death including apoptosis, has also been reported [73]. The current review provides further insight into the physiological functions of JNK3, such as development, regeneration and differentiation/reprogramming.

### 3.1. The Distribution of JNK3 in Neuronal Tissue

The monoclonal antibody 3F12, which recognizes human JNK3, stained a subpopulation of pyramidal neurons in the CA4, CA1, subiculum regions of the hippocampus and layers 3 and 5 of the neocortex, in normal human brains [23,25]. Northern blotting indicated that the distribution of mRNA was restricted to a subset of projection neurons in the nervous system, whereas in non-neuronal tissues, only the testes and kidneys showed expression and that too at low levels.

Immunohistochemistry using MAb 3F12 indicated that the JNK3 protein was expressed in the neurons of the hippocampus, cortex, cerebellum, brain stem and spinal cord of adult mice [24]. A developmental study involving northern blotting showed that the expression of mouse JNK3 mRNA begins at E12.5 and continues throughout development into adulthood [24]. In situ hybridization of mouse fetal tissue revealed that no signals were detected in embryos from days E8 through El0. However, JNK3 mRNA expression was detected on day E11.5. Furthermore, mRNA expression was detected in both central and peripheral nervous systems by E17.5. In the central nervous system, mRNA expression of JNK3, which was first detected in the rostral spinal cord and rhombencephalon at E11.5, was evident throughout the telencephalon by E12.5-13. JNK3 mRNA was expressed in post-mitotic neurons of the cortical plate, whereas no expression was detected in dividing cells of the paraventricular zone. By E17.5, the expression of JNK3 mRNA was also detected in the neurons of dorsal root and sensory ganglia and at lower levels in the neurons of myenteric and cardiac plexuses as well as in post-ganglionic parasympathetic neurons of the vagus nerve in the developing heart [24]. Immunocytochemical analysis of developing mouse embryos revealed that, by E7.5, JNK3 was co-expressed with JSAP1 in the embryonic and extra-embryonic ectoderm, as well as in the extra-embryonic endoderm. Co-expression of β-Tubulin III, JNK3, and Wnt1 with JSAP1 was observed in the neuroectoderm. Western blotting confirmed high level expression of JNK3 and JSAP1 at E6.5 and E7.5. [74].

In situ hybridization revealed that JNK3 and JNK2 mRNAs were mainly expressed in adult rat brains. JNK3 signal was found in neuronal-like cell bodies of the cerebral cortex, hippocampus and diencephalic areas. JNK2 signal was generally localized in the same brain areas as JNK3, whereas JNK1 signal was low and restricted to the endopiriform nucleus and the medial habenula. In newborn rats, JNK3 mRNA expression appeared in the same brain areas as in those of adults. In P9 and P12, the mRNA expression of JNK3 was decreased and gradually returned from P15 to early days after birth [75].

### 3.2. In Vivo Function of JNK3 in Neuronal Tissue

Generation of JNK3 knockout mice was first reported by Yang et al. They created a targeting vector to replace an internal 4 kb Mscl–Spel Jnk3 genomic fragment with a PGKneo cassette. The deleted region encompassed one and a half exons encoding amino acids 211–267 of Jnk3, including the dual phosphorylation motif Thr-Pro-Tyr, which is characteristic of the JNK family and is important for protein kinase activity. Chimeric mice were generated by injecting these ES cells into C57BL/6 blastocysts. Heterozygotes (+/−) were intercrossed to generate homozygous mutant mice (−/−). Northern blotting, RT−PCR and protein kinase assays confirmed that the *Jnk3* (−/−) mice were deficient in JNK3 expression and activity, demonstrating that the targeted disruption of *Jnk3* resulted in a null allele. In *Jnk3* (−/−) mice, there was no developmental abnormality, as indicated by fertility, normal size and normal structural organization/cellular composition of the brain [76].

Mice deficient in a single JNK gene were viable and bred normally without apparent morphological abnormalities, suggesting that functional redundancy may exist in the JNK family. Thus, *Jnk1*, *Jnk2*, and *Jnk3* null mutant mice were crossed to generate compound mutants that were deficient in any two members of the JNK family. Although both JNK1/JNK3 or JNK2/JNK3 double knockout mice survived normally, JNK1/JNK2 double knockout mice were embryonic lethal and had severe dysregulation of apoptosis in the brain [77].

Under physiological conditions, the number of GFAP+/Sox2+ cells in the subgranular zone (SGZ) of the hippocampus was lower in Jnk3 knockout mice compared with that in WT. The first Doublecortin+/calretinin+ differentiated neuron subtype in the SGZ was significantly decreased in *Jnk3*^−/−^ CT mice compared to WT. Immunoreactivity of calbindin was higher in mature granule cells in adult DG of *Jnk3*^−/−^ CT mice compared with that in WT. These observations suggested that JNK3 is involved in the regulation of adult neurogenesis [78].

The behavior of JNK1, JNK2 and JNK3 single knockout mice was examined via elevated plus maze, open field, novel object recognition memory and Morris water maze tests [79]. The elevated plus maze test, which evaluated anxiety, revealed that although JNK3 KO and JNK2 KO mice were less active than WT and JNK1 KO mice, there was no difference in anxiety behavior. The open field test evaluates anxiety-like and locomotion behaviors. Mice preferentially move around the periphery of equipment when they are placed in an open field of a novel environment. Time spent in the central area is inversely correlated to their anxiety associated proneness. Although no difference was observed between WT, JNK2 KO and JNK3 KO mice, JNK1 KO mice were more explorative than the other genotypes. The novel object recognition memory test is a parameter of non-hippocampal learning efficiency. The recognition index, total time spent with all objects, total distance moved, and non-hippocampal memory consolidation was not affected by genotype. However, total time spent in not-moving was significantly less in JNK1 KO compared to that of WT and JNK3 KO, suggesting JNK1 KO mice showed higher levels of exploration and locomotion than WT, JNK2 KO or JNK3 KO. The Morris water maze test (a training phase over 3 d followed by a test day) was performed for hippocampal learning. In the training phase, JNK3 KO mice spent more time in the border zone than JNK1 KO mice. In the test phase, JNK2 KO mice spent more time in the border zone than JNK1 KO mice, but no significant effect was detected between WT and JNK3 KO mice, suggesting that JNK1 KO mice were more explorative than the other genotypes. In summation, single JNK KO provokes a phenotypic explorative behavior without affecting memory consolidation. JNK1 KO mice showed the greatest extent of behavioral change compared to other genotypes. On the other hand, JNK2 KO and JNK3 KO mice revealed a tendency to behave in a manner opposite to that of JNK1 KO mice.

### 3.3. In Vivo Function of JNK3 in Neuronal Regeneration

Damage to the sciatic nerve induces structural reorganization of axons within the central projection area of dorsal root ganglion (DRG) neurons. MAP1B phosphorylation is a marker of axonal sprouting and regeneration in the adult nervous system. Fibers in lamina II ipsilateral to the sciatic nerve lesion showed an increase in phosphor-JNK intensity, which was co-localized with phosphor-MAP1B. These results demonstrated that DRG neurons exhibit an increase in activated JNK in the regenerative state in cytoplasm. Phosphor-JNK expression was also observed in the nucleus as well as in the cytoplasm of primary cultures of adult mouse DRG neurons, plated for 48 h. Treatment of neurons with the JNK inhibitor, SP600125, 4 h after plating, resulted in the inhibition of neuritogenesis. Although the rate of neuritogenesis in the DRG neurons of JNK3 KO mice was reduced, neurite length remained unchanged [80]. On the other hand, neuritogenesis and neurite length of *Jnk1*^−/−^ and *Jnk2*^−/−^ neurons showed a significant decrease, suggesting that the JNK1 and JNK2 isoforms were dominant in cytoskeleton reorganization and neurite regeneration. In mouse DRG neurons, deletion of JNK3 slightly reduced neurite length, but deletion of either JNK1 or JNK2 led to a significantly greater reduction than that caused by the deletion of JNK3, suggesting that JNK3 may be a promising target for suppression of apoptosis with minimum adverse effects on neurite regeneration [81].

The effect of JNK3 on axonal regeneration following facial nerve axotomy has been reported [82]. Although homozygous deletion of JNK2 had no effect, deletion of both JNK3 and JNK1 caused a significant delay in functional recovery. Deletion of JNK3 and JNK1 did not affect reconnection rates, extent of axonal elongation, neuronal loss or neuronal cell size. JNK3 null mutants showed a significant reduction in the immunoreactivity of the adhesion molecule CD44, but levels of the neuropeptide CGRP and the transcription factor ATF3 were not affected. Deletion of JNK1 or JNK2 had no effect on immunohistochemical neuronal injury-associated markers. In *jun*ΔS mutants, the elongation of axons containing neuropeptides, CGRP or galanin, was reduced. However, the extent of axonal elongation following nerve crush was not observed in homozygous JunAA mutant mice. Neuronal c-Jun is necessary for axonal elongation, but JNK-induced activity may not be required. It is most likely that functional recovery depends on the gradual formation of a non-neuronal cellular bridge regulated by JNK3.

### 3.4. Ex Vivo and In Vitro Function of JNK3 in Cultured Neurons

Acute and chronic effects of hyperosmotic stress and stimulation of glutamate receptors on JNK3 expression in primary cultures of rat magnocellular neuroendocrine cells were examined [83]. Acute osmotic stimulation (1 h) with hypertonic NaCl resulted in an increase in JNK3 immunoreactivity in the perikarya and processes. During stimulation, cell size acutely decreased within 2 min but recovered to normal size over a period of 20 min. Long period hyperosmotic stimulation (24 h) provoked a slight increase in the intensity of JNK3. Osmotic stimulation also induced the phosphorylation of c-Jun, suggesting that hyperosmolality increased the activation of JNKs. The effects of the osmotic stimulation-induced increase in JNK3 intensity were attenuated by TTX. A separate analysis of small and large subsets of neurons revealed that osmotic stimulation increased JNK-3 staining in small neurons but not in large neurons. Concerning the role played by glutamate receptors, acute stimulation with the metabotropic glutamate receptor agonist, 1S3R ACPD, increased JNK3 staining. Long period stimulation with the ion channel glutamate receptor agonist, NMDA, induced a small but nonsignificant increase in JNK3 staining, but significantly raised the hyperosmotic effect exerted on JNK3 staining. These observations suggested that JNK3 was involved in the signal transduction cascade associated with cell volume regulation.

Neurite outgrowth in primary midbrain dopaminergic neuron cultures of the mesencephalon floor of embryonic day 14 Wistar rats was analyzed using a scratch assay. The JNK inhibitor SP600125 attenuated neurite outgrowth. Neurite outgrowth was decreased in the total population of JNK3 siRNA transfected neurons, as well as in the dopaminergic neuron subpopulation. The effects exerted by the transfection of JNK3 siRNA was rescued by ectopic JNK3 expression. Cellular viability was also reduced by the transfection of JNK3 siRNA. Considered together, these findings imply that JNK3 contributes to neuronal regeneration and cellular survival in damaged dopaminergic neurons [84].

Removal of nerve growth factor (NGF) increased JNK3 activity in the sympathetic neurons of the superior cervical ganglia of 1-day-old Sprague–Dawley rats, leading to increased phosphorylation of c-Jun. A shift in SCG10 bands, observed 6–12 h following NGF removal by western blotting, was attenuated by alkaline phosphatase treatment. A shift in SCG bands was also observed in mutant SCG10 expressed in COS-7 cells. These observations suggested that JNK3 induced phosphorylation under cellular stress conditions [35].

Axonal complexity of neurons overexpressing p54 JNK3α2 CS was increased, as indicated by higher branch numbers and longer total axonal lengths, compared with neurons overexpressing WT p54 JNK3α2. Axonal filopodia in neurons overexpressing p54 JNK3α2 CS showed higher motility. Thus protein palmitoylation has been newly recognized as a mechanism underlying isoform-specific regulation of JNK3. The above findings also suggest a potential role for JNK3 palmitoylation in modulating axonal branching. However, phosphorylation levels of wild type JNK3 (JNK3 WT) were similar to those of palmitoylation-deficient mutant JNK3 (JNK3 CS) in heterologous cells challenged with or without osmotic stress. This implied that palmitoylation may not be involved in JNK3-regulated stress response. Palmitoylation of p54 JNK3α2 did not result in enrichment of the lipid raft marker caveolin-1. However, an increase in JNK3 CS was observed in the high-density Triton-X100-insoluble fraction enriched with the actin-associated cytoskeleton. JNK3, which was detected in the Triton-insoluble fraction of neurons and HEK293 cells expressing GFP-JNK3, decreased in the presence of actin depolymerizers, cytochalasin D or latrunculin A. The amount of JNK3 in the Triton-insoluble fraction of HEK293 cells co-expressing PAT zD15 was decreased. Palmitoylation of JNK3 in five DIV hippocampal neurons treated with Wnt7a was also decreased. Wnt 7a also induced the translocation of JNK3 to the Triton-insoluble cytoskeleton fraction. Wnt 7a induced axonal branching in neurons overexpressing WT JNK3 but not in JNK3 CS-transfected neurons. These observations suggested that palmitoylation of JNK3 regulates axonal development [66].

### 3.5. In Vitro Function of JNK3 in Cellular Models of Neuronal Differentiation and Reprogramming

The use of in vitro model systems in neuroscience has provided valuable information. The mRNA expression of JNK isoforms has been reported in several neuronal differentiation cell models, such as rat PC12, mouse Neuro-2A and human SHSY5Y cells [24,85]. Undifferentiated mouse Neuro-2A cells expressed JNK1, JNK2 and JNK3 mRNA and proteins, whereas rat PC12 cells expressed only JNK1 and JNK2 mRNA and proteins. The expression of mRNA and proteins in human SH5Y5Y cells was inconsistent, where only JNK2 mRNA expression was detected via RT-PCR, in contrast to protein expression of JNK1, JNK2 and JNK3 detected via western blotting [85]. Expression of JNK3 mRNA is not inducible during neuronal differentiation. In PC12 and Neuro-2A cells, several neuronal differentiation inducers (e.g., dibutyryl cAMP- or retinoic acid treatment) did not exert an effect on JNK3 mRNA expression [24].

In PC12 cells, stimulation by NGF decreased mRNA expression of the A_2A_ adenosine receptor, which contributes to physiological processes in both central and peripheral nervous systems, including vasodilation, respiratory depression, wakefulness, and spontaneous locomotor activity [86]. NGF failed to extend the neurites in PC12 cells transiently transfected with dominant negative JNK3 (K55A). NGF-induced down regulation of mRNA expression of A_2A_ adenosine receptor was attenuated in PC12 cells expressing JNK3 (K55A). When dominant negative JNK3 (K55A) expressing PC12 cells were co-incubated with the MEK inhibitor PD98059, downregulation of A_2A_ adenosine receptor mRNA was completely inhibited [86]. Radioligand binding assay confirmed that NGF-induced down regulation of A_2A_ adenosine receptor expression was recovered by the transfection of dominant negative JNK3 (K55A) and the MEK inhibitor, PD98059. NGF stimulation also induced the expression of neurofilament light chain (NF-L), a marker for neuronal differentiation, in PC12 cells. Zentrich et al., reported that MKK7β1-p46 JNK3α1 fusion proteins induced the NF-L-Luc reporter signal. Although NGF-induced JNK activation was obscure, a kinase-inactive form of MKK7 inhibited NGF-induced NF-L-luciferase activity. The CRE/ATF site deletion mutant completely abolished NGF induction of NFLC promoter activity. EMSA revealed that a transcriptional factor complex, including CREB, c-Jun and JunD, binds to the CRE/ATF-site in the NF-L promoter. Analysis of the effect of transfected c-Jun, JunD, CREB and ATF2 on NGF-induced luciferase activity demonstrated the dominant contribution of c-Jun to NGF-induced NF-L expression. These observations suggested that NGF induced the activation of JNK/c-Jun, resulting in the expression of NF-L via promoter activity in the CRE/ATF site [87].

Transfection of p54 JNK3α2 into serum-starved PC12 cells with NGF increased phosphorylation of p54 JNK3α2 and c-jun. However, no differences between the phosphorylation levels of JNK1, JNK2 and ATF-2 of un-transfected and transfected cells were detected. NGF-induced expression and phosphorylation of the neurofilament heavy chain (NF-H) was enhanced in PC12 cells expressing p54 JNK3α2. NGF-induced morphological changes (the number and length of neurites) were also accelerated by the ectopic expression of p54 JNK3α2. These observations suggest that a substrate switch between ATF-2 and c-Jun contributes to the differentiating-regenerating potential of p54 JNK3α2. Although p54 JNK3α2 signalosome may account for this substrate switch, the underlying mechanisms are yet to be elucidated [88].

MKK7-JNK3 fusion protein induced c-jun transactivation in PC12 cells was attenuated by transient expression of myristoylated Akt, suggesting that Akt acts was a negative regulator of JNK3 activation [89].

The growth of JSAP1-null (*Jsap1*^−/−^) ES cells was faster than that of wild type or parental *Jsap1*^+/−^ ES cells. The embryo bodies (EBs) in JSAP1−null ES cells was smaller than those of wild type cells in the absence of retinoic acid (RA). The treatment of JSAP1−null EBs with RA resulted in the formation of much smaller EBs. Neurites observed in JSAP1-null EBs in the absence of RA were longer compared to those observed in wild type. By contrast, RA-induced neurite elongation was poor in JSAP1-null EBs. The expression levels of Emx2, Otx1 En2, Wnt1 and Pax5 were low in both undifferentiated wild type and JSAP1-null ES cells, but Pax2 expression was high. An increase in the Pax5 mRNA expression level was observed in wild type early EBs. Significant increases in Emx2, Otx1, Wnt1 and Pax5 mRNA expression levels were observed in late JSAP1-null EBs without RA [72]. RA induced the expression of Emx2, Otx1 En2, Wnt1, Pax2 and Pax5 in wild type, but not in JSAP1-null ES cells. The expression of MKK4, MKK7 and JNK3 increased during RA-induced EB formation of wild type, but not JSAP1-null ES cells. JSAP1-null EBs showed hyperplastic proliferation of ectodermal epithelium and formed multitubular structures. A decrease of JNK3 expression and an increase in β-tubulin III and Wnt1 were observed in hyperplastic ectodermal layers of JSAP1-null EBs, [74]. Thus, JSAP1 affected the expression of JNK3, as well as RA-induced neuronal differentiation.

Investigating the regeneration of the central nervous system poses challenges. Cell transplantation therapy presents a promising approach that enables functional recovery from a damaged central nervous system. Previous studies have indicated that adipose tissue provides a cellular source for studying cellular reprogramming. Dedifferentiated fat cells (DFATs), fibroblast-like cells derived from mature adipocytes, have shown properties, such as abundance, isolation method, robust proliferation capacity and homogeneity, that are suitable for studying cellular reprogramming. When canine DFATs were treated with all-trans retinoic acid (ATRA), the expression of neuronal markers (NF-H, MAP2, Ascl1, NeuroD2 and NGFR) increased. Neuron-like cells showed TTX-sensitive action potential and voltage-dependent Ca^2+^ influx. Optical imaging of presynaptic terminal activity and detection of neurotransmitter release indicated that neuron-like cells exhibited GABAergic neuronal properties. Genome-wide RNA-sequencing analysis showed that the transcriptome profile of canine DFATs is effectively reprogrammed towards that of cortical interneuron lineage. Thus, neuron-like cells from canine DFATs may also provide a powerful tool for translational research in cell transplantation therapy, in vitro disease modeling and screening of drugs for neuronal diseases. The mechanism underlying neuronal reprogramming was investigated using unbiased screening via RNA-seq-based pathway analysis. Pathway analysis and inhibitor screening indicated the involvement of JNK signaling. In knockdown experiments, transfection with JNK3 siRNA attenuated ATRA-induced NGFR mRNA expression, whereas JNK1, JNK2 or scramble siRNAs did not, suggesting that JNK3 contributes to the ATRA-induced neuronal reprogramming process [5].

### 3.6. In Vitro Function of JNK3 in Other Types of Cells

The mRNA and protein expression of JNKs, including JNK3, was detected in rat primary cultured astrocytes. Knockdown experiments using isoform specific siRNA showed that JNK3 phosphorylation was induced by Trag-induced proteinase-activated receptor-1 (PAR-1) activation and P2AP-induced PAR-2 activation. PAR-1-induced chemokine growth-regulated oncogene/cytokine-induced neutrophil chemoattractant-1 (GRO/CINC-1) release was attenuated by JNK3 siRNA. The effect of JNK3 siRNA was enhanced by co-transfection with JNK2 siRNA. JNK3 siRNA failed to attenuate PAR-2-induced GRO/CINC-1 release, whereas JNK1 siRNA clearly inhibited PAR-2-induced GRO/CINC-1 release. These observations suggest that PAR-1-induced GRO/CINC-1 release was mediated by JNK3 and JNK2 activation [90].

The involvement of JNKs in the contractility of mouse blood vessels has been reported [91]. Contractions induced via KCl were increased in the arteries of JNK2/3 double knockout mice. The maximal contraction induced by phenylephrine or noradrenaline was significantly enhanced in JNK2/3 knockout arteries. Inhibition of NOS by Nw-nitro-l-arginine attenuated noradrenaline-induced vasoconstriction enhancement in JNK2/3 double knockout mice. In summary, genetic deletion of JNK2/3 in mice results in altered contractility of carotid arteries. It is most likely that the enhancement of vasoconstriction depends on the function of smooth muscles in blood vessels. Mouse hindlimb ischemia (HLI) models lacking JNK3 showed significantly enhanced blood flow recovery in response to HLI compared with WT controls, suggesting that ischemia-induced JNK3 expression in the peripheral nerves played a role in the blood flow recovery process, by regulating pro-angiogenic genes [92].

In insulin producing cells (INS-1E cell line), JNK3 silencing markedly decreased the protein expression of insulin receptor substrate 2 (IRS2), which mediates the activation of PI3K/Akt signaling and regulates β-cell proliferation, survival, insulin synthesis and secretion. Thus, JNK3 maintains the IRS2/Akt2 signaling module required to preserve β-cell function and mass [93]. The glucagon-like peptide-1 receptor agonist, exendin 4, induced JNK3 expression in isolated human islet and INS-1E cell lines. The rate of apoptosis was increased in INS-1E cells treated with a cytokine cocktail containing rat IL-1β, mouse TNFα and rat IFNγ. In cells transfected with scramble siRNA, exedin 4 attenuated cytokine cocktail-induced apoptosis in cells transfected with JNK3 siRNA, but failed to prevent cytokine-induced apoptosis. These observations suggest that JNK3 contributes to the antiapoptotic effects of exendin 4 [94]. The activity of dual leucine zipper-bearing kinase (Dlk, MAP3K12) in highly proliferative neonatal rat islet cells was increased. Dlk overexpression in β-cell cytoplasm was associated with increased JNK3 activity. Dlk interacted with and induced the activation of JNK3, which stimulated postnatal β-cell replication via the expression of cyclin genes, *Ccnd1* and *Ccnd2*. Silencing of Dlk or Jnk3 in neonatal islet cells attenuated primary β-cell replication and the expression of *Ccnd1* and *Ccnd2*. Cytoplasmic DLK expression was also observed in healthy human islets. Expression of DLK mRNA was correlated with an increase in JNK3, CCND1 and CCND2 mRNA levels. These observations suggested that activation of JNK3 signaling by Dlk contributed to islet β-cell proliferation during postnatal development [95].

In the reprogramming of cardiac fibroblasts into cardiomyocytes (CMs), a cocktail of three genes, *FoxM1*, *Id1*, and *Jnk3*-shRNA (FIJs), was found to induce increases in α-MHC^+^/Ki67^+^ and H3P^+^ populations [96]. Furthermore, the ability of FIJs to enhance adult CM proliferation in vivo was demonstrated by direct injection into the hearts of 10-week-old mice. The mRNA expression of mitosis markers, *Aurkb*, *Mad2L1* and *Plk1*, was increased in CMs treated with FIJs. Expression of Cdk4 mRNA was upregulated in Id1-treated CMs, whereas FoxM1 upregulation and *Jnk3*-shRNA were most effective at increasing Cdk2 mRNA expression. Indeed, treatment with combined FIJs effectively increased both Cdk4 and Cdk2 mRNA expression. Each of the three components of FIJs treatment was able to significantly enhance Cdk1 expression. An inhibitor of CDK4, p16, was downregulated only in treatment groups containing Id1. The expression of the CDK2 inhibitor p27 was clearly reduced in CMs treated with *Jnk3*-shRNA. Furthermore, p21 was significantly inhibited by FoxM1. Therefore, it is most likely that FIJs treatment regulates CM proliferation via the inhibition of CDK inhibitors, which in turn induces CDK expression [96].

Cellular reprogramming studies indicated that JNK3 most likely promotes cellular reprogramming into neurons and neuroendocrine cells and attenuates cardiac lineage reprogramming. However, its role in the selective reprogramming process remains to be elucidated.

## 4. Conclusions and Perspectives

Although JNK3 also plays a crucial role in pathological phenomena, the current review focused on the physiological function of JNK3. Many new substrates of JNK3, as well as of proteins interacting with JNK3, have been identified. However, the cellular context-dependent function of JNK3 remains to be resolved. In neurons, an increase in [Ca^2+^]_i_ associated with action potential invasion induces a variety of specific neuronal functions including neurotransmitter release, cellular growth, excitability, synaptic plasticity and transcriptional regulation [97]. In addition, the cell type-specific features in calcium dynamics probably contribute to the different role in the neuronal network [98]. In the previous study, it has been reported that the calcium signaling contributed to the activation of JNK3 in pathophysiological conditions such as ischemia- or soman-induced neuronal death [99,100]. However, the role of calcium signaling in JNK3 activation in physiological condition has been unclear. In our previous report, JNK3 contributed to ATRA-induced neuronal reprogramming in canine dedifferentiated fat cells, but the Gq inhibitor YM254890 had no effect [5]. On the other hand, Gq/11-coupling receptor AT1a receptor induces the activation of JNK3, suggesting that calcium signaling regulates JNK3 activation in a cellular context dependent manner [40]. We expect that the progress made in the omics approach and single cell technologies would address the basic molecular mechanisms that underly this cellular context dependent regulation and provide further insight into the biological functioning of JNK3. Furthermore, the biological significance of neuronal specific distribution as well as certain functions of JNK3 are yet to be uncovered. In conclusion, JNK3 is a key player in neuronal cell biology as reflected by studies conducted on neuronal function in the developing and adult brain, which revealed that JNK3 activation contributes to neuronal differentiation and reprogramming in cellular models. An understanding of mechanisms underlying JNK3 signaling would be important for enhancing regeneration and cell transplantation therapies targeting injured central nervous tissues.
